# Surveillance of *Borrelia miyamotoi*-carrying ticks and genomic analysis of isolates in Inner Mongolia, China

**DOI:** 10.1186/s13071-021-04809-z

**Published:** 2021-07-17

**Authors:** Gaowa, Wulantuya, Kozue Sato, Dan Liu, Yunhong Cui, Xuhong Yin, Lihua Zhang, Hong Li, Tingfu Wang, Rongxin Liu, Lijing Wu, Saixia Lu, Ting Gao, Zitong Zhang, Minzhi Cao, Guodong Wang, Chunpu Li, Dacheng Yan, Norio Ohashi, Shuji Ando, Hiroki Kawabata

**Affiliations:** 1 Inner Mongolia Key Laboratory of Tick-Borne Zoonotic Infectious Disease, Department of Medicine, College of Hetao, Bayan Nur, 015000 Inner Mongolia Autonomous Region China; 2 Department of Bacteriology-I, National Institute of Infectious Diseases, Toyama 1-23-1, Shinjuku-ku, Tokyo, 162-8640 Japan; 3 Bayan Nur Centers for Disease Control and Prevention, Bayan Nur, 015000 Inner Mongolia Autonomous Region China; 4 Hulunbuir Centers for Disease Control and Prevention, Hulunbuir, 021000 Inner Mongolia Autonomous Region China; 5 Laboratory of Microbiology, Department of Food and Nutritional Sciences, University of Shizuoka, Shizuoka, Shizuoka 422-8526 Japan; 6 Department of Virology, National Institute of Infectious Diseases, Shinjuku-ku, Tokyo, 162-8640 Japan

**Keywords:** *Ixodes persulcatus*, *Borrelia miyamotoi*, MLSA, Inner Mongolia

## Abstract

**Background:**

*Borrelia miyamotoi* is a newly described relapsing fever spirochete transmitted by ixodid tick species. Little is known about the prevalence of *B. miyamotoi* infections in humans and ticks in Inner Mongolia, China. Therefore, we investigated the prevalence of *B. miyamotoi* in *Ixodes persulcatus* ticks, and we aimed to isolate*B. miyamotoi* from *I. persulcatus* from four regions of Greater Khingan, Inner Mongolia, China.

**Methods:**

From May to June each year during the period 2016–2019, host-seeking adult *I. persulcatus* ticks were collected from vegetation. Genomic DNA was prepared from half of each tick body for PCR template, and the remaining half was used to cultivate *B. miyamotoi* in BSK-M medium. We employed quantitative real-time PCR (qPCR) to detect *Borrelia* DNA in the ticks and to calculate the prevalence of *B. miyamotoi* and infections with other borreliae. For characterization of the isolated *B. miyamotoi*, we performed draft genome sequencing and multilocus sequencing analysis (MLSA).

**Results:**

A total of 2656 adult *I. persulcatus* ticks were collected. The overall prevalence of relapsing fever (RF) borreliae in ticks was 5.0% (134/2656) and that of Lyme disease (LD) borreliae was 43.8% (1164/2656). Co-infection with RF and LD borreliae was observed in 63 ticks (2.4%). Ticks that were positive for RF borreliae by qPCR were subjected to glycerophosphodiester diester phosphodiesterase gene (*glpQ*) PCR amplification and sequencing, through which we identified the RF borrelia specimens as *B. miyamotoi*. Furthermore, the *B. miyamotoi* strain Hetao-1 was isolated from *I. persulcatus*, and a draft genome sequence was obtained from the isolate. Sequencing determined the strain Hetao-1 genome to be approximately 906.1 kbp in length (28.9% average GC content), and MLSA identified the strain as ST633, which has previously been reported in Japan and Mongolia.

**Conclusion:**

We detected *B. miyamotoi* from *I. persulcatus* ticks collected in Inner Mongolia, and successfully isolated a *B. miyamotoi* strain. To our knowledge, this is the first study to culture a *B. miyamotoi* isolate from China. The data on the prevalence of *B. miyamotoi* and other borreliae in *I. persulcatus* ticks will be fundamental for future epidemiological studies of *B. miyamotoi* disease in Inner Mongolia.

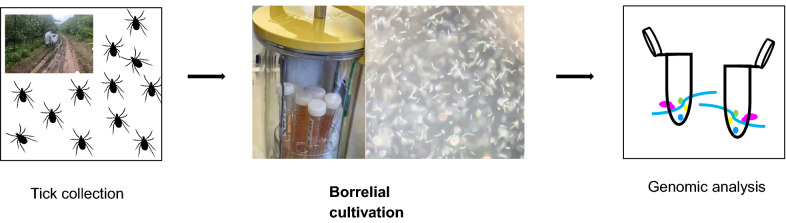

## Background

*Borrelia miyamotoi* and other genetically related relapsing fever (RF) borreliae are transmitted by *Ixodes* ticks, which are also vectors for the agents of Lyme disease [1, 2]. *B. miyamotoi* was first discovered from the tick *I. persulcatus* and the rodent *Apodemus argenteus* in Japan [3] and is considered an emerging pathogen in humans [4]. The spirochete *B. miyamotoi* has been shown to cause an infectious disease in humans now referred to as *B. miyamotoi* disease (BMD), and has been reported in Russia, the United States, several European countries, Japan, and China [4–12]. BMD manifests as a high fever (up to 40 °C), fatigue, headache, myalgia, chills, nausea, and arthralgia, and meningoencephalitis has been reported in immunocompromised patients [8, 13]. To date, *B. miyamotoi* has been found in *I. scapularis* and *I. pacificus* ticks in North America [14, 15], *I. ricinus* in Europe [16], and *I. persulcatus*, *I. ovatus*, and *I. pavlovskyi* in Asia [17, 18].

In China, cases of Lyme disease (LD) have been reported in Greater Khingan and Lesser Khingan in the northeast, where the principal LD vector, *I*. *persulcatus*, is abundant [19]. *I*. *persulcatus* has been confirmed to also carry *B. miyamotoi* in northeastern China, and BMD was reported in the region in 2018 [12]. However, there has been a dearth of regional surveys of *B. miyamotoi* infection in tick populations. Not only do the epidemiology and prevalence in China remain unclear, but also the genetic characteristics of the resident *B. miyamotoi*, due to difficulty in cultivating the bacteria. This basic information on the prevalence of *B. miyamotoi* infection in ticks, and the genetic characterization of the pathogen, are urgently required for risk assessment of BMD in northeastern China.

The Greater Khingan region in northeastern China offers favorable environmental conditions for the survival and proliferation of *I. persulcatus*. In this area, tick bites in people are common, and human ixodid tick-borne infections, including those caused by LD borreliae and tick-borne encephalitis virus (genus *Flavivirus*), are endemic and transmitted by the same tick species [20]. However, some febrile patients have a history of tick bite, and despite the possibility of tick-borne infection, laboratory diagnosis has not been able to identify the infectious agent. In this study, large-scale surveillance for *B. miyamotoi* was conducted in Greater Khingan to estimate the infection rate of host-seeking adult *Ixodes* ticks. The tick-derived isolates of *B. miyamotoi* discovered in this study were subjected to molecular analysis to characterize their genetic profile. The resultant field and laboratory data will serve as a baseline for research aiming to understand the epidemiology of *B. miyamotoi* in Inner Mongolia, China.

## Methods

### Study area

**Fig. 1:**
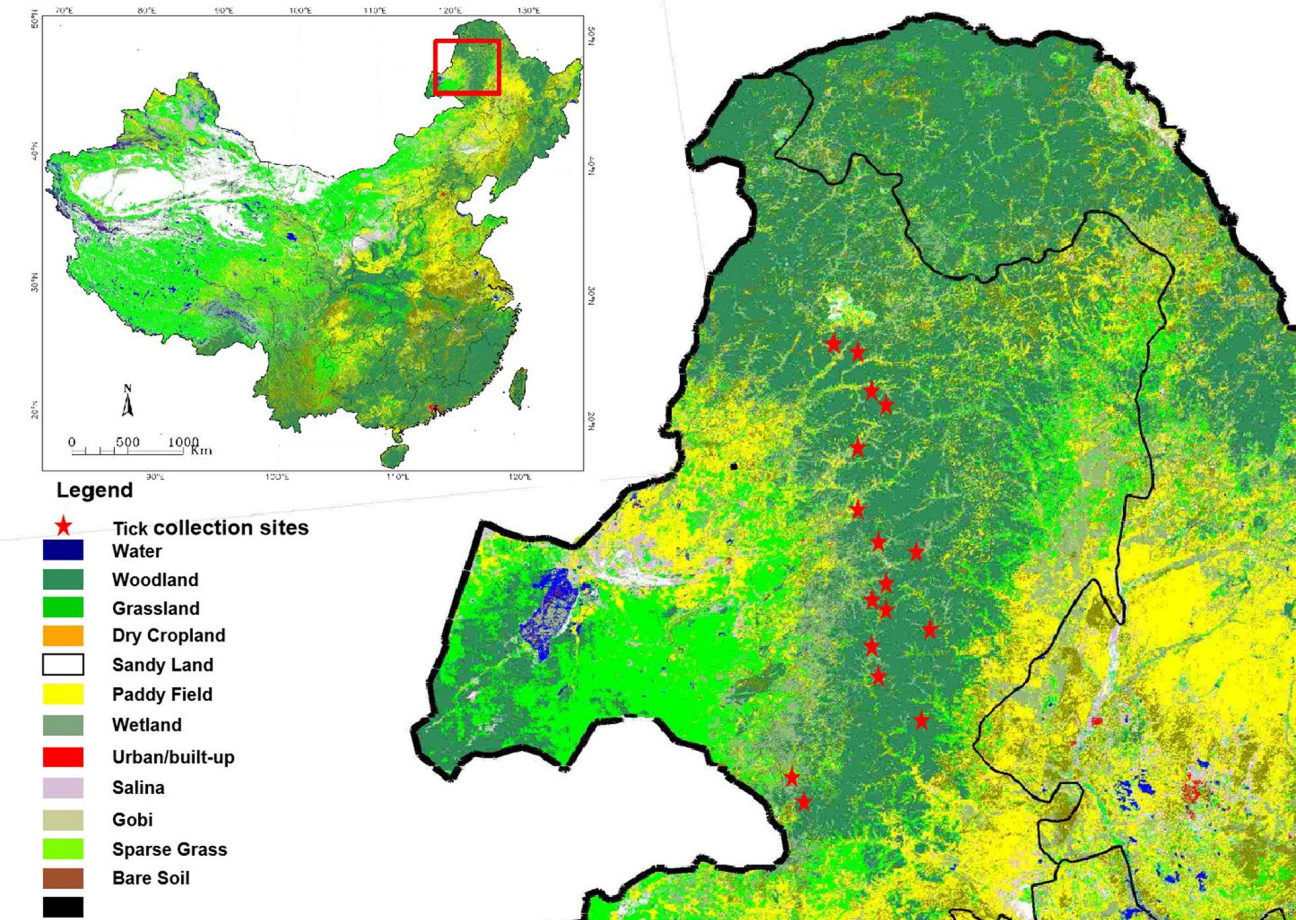
Tick collection areas in this study. Ticks were collected from the areas shown by red stars

The tick samples in this study were collected in different forested areas throughout Greater Khingan in Hulun Buir City of Inner Mongolia, northeastern China (Fig. 1) [21]. The Greater Khingan forest region of Inner Mongolia is in the northernmost area of the Greater Khingan Mountains, accounting for 46% of the total area, with geographical coordinates of 119° 36′ 30″ to 125° 24′ 00″ E and 47° 03′ 40″ to 53° 20′ 00″ N. The main habitat is primeval forest at an altitude of 250–1745 m, an average annual temperature of −3.5 °C, and annual precipitation of 300–450 mm. In these areas, no specific permission was required for the collection of ticks, and this study did not involve endangered or protected species.

### Tick collection, DNA extraction, and borrelial cultivation

From May to June each year from 2016 through 2019, host-seeking adult ticks were collected by flagging from vegetation. The collected tick samples were placed in collection tubes, which were classified and numbered according to the sampling time and place. *I. persulcatus* ticks were identified by morphological characteristics [22]. Ticks were washed with 0.1% sodium hypochlorite and 75% ethanol containing povidone iodine for 5 min, washed again with 3% hydrogen peroxide for 5 min, and then rinsed with sterile water. A genomic DNA PCR template was prepared from half of each tick body according to Yamazaki-Matsune et al. [23]. The remaining half was used to cultivate *B. miyamotoi* in modified Barbour–Stoenner–Kelly medium (BSK-M: using minimal essential medium alpha [Bio West, Germany] as a substitute for CMRL-1066) under microaerophilic conditions [17, 24]. The tick samples that were positive for RF borreliae and negative for LD borreliae on qPCR were cultivated at 30 °C for 4 weeks, and the growth of spirochetes was examined by dark-field microscopy every 2 weeks.

### Detection of borrelial DNA from ticks

Tick lysates were subjected to qPCR assay to detect borrelial infection. The assay was designed by Barbour et al. to specifically detect RF borreliae, including *B. miyamotoi*, and LD borreliae in tick lysates by multiplex qPCR targeting the 16S rRNA gene (*16S rDNA*) [25]. To enable the detection of most *Borrelia* spp., common primers targeted conserved sequences, and specific DNA probes conjugated to non-fluorescent quencher (NFQ) and minor groove-binder architectural protein (MGB) were designed. The two probes were labeled with either the fluorescence reporter group FAM or VIC, and the multi-qPCR reaction system was able to simultaneously detect RF and LD borreliae. The forward and reverse primers were 5′-GCTGTAAACGATGCACACTTGGT-3′ and 5′-GGCGGCACACTTAACACGTTAG-3′, respectively. The corresponding dye-labeled probes, FAM-TTCGGTACTAACTTTTAGTTAA-NFQ-MGB and VIC-CGGTACTAACCTTTCGATTA-NFQ-MGB, were purchased from Applied Biosystems (Foster City, CA). The qPCR was performed using Premix Ex *Taq* (Probe qPCR, Takara Bio Inc., Shiga, Japan) according to the manufacturer’s instructions and run on a Bio-Rad CFX96 system with 42 PCR cycles. Quality control in the nucleic acid amplification method in this study was performed as previously reported by Espy et al. [26]. A negative (blank) control was used in all qPCR runs. Plasmid DNA was used as a positive control for qPCR as previously reported by Takano et al. [17]. For conventional PCR, genomic DNA extracted from *B. miyamotoi* strain HT31 was used as a positive control.

### Conventional PCR and phylogeny reconstruction using *glpQ* sequences

To confirm the qPCR results, we performed conventional PCR on the tick-derived isolates. Ticks that were found to be RF-DNA-positive by qPCR were subjected to glycerophosphodiester diester phosphodiesterase gene (*glpQ*) analysis with PCR-based DNA sequencing [27] using primers purchased from Nanjing GenScript Biological Technology Company: forward primer (*glpQ*-F), 5′-CATACGCTTATGCYTTRGGMGCTGA-3′, and reverse primer (*glpQ*-R), 5′-GCAACCTCTGYCATACCTTCTTSTG-3′. The amplicon was approximately 610 bp in length. The reaction conditions of the first PCR were 3 min at 94 °C, then 30 cycles of 30 s at 94 °C, 30 s annealing at 53 °C, 30 s at 72 °C, and finishing with 5 min at 72 °C. In the second PCR, the annealing temperature was changed to 55 °C. We employed the Blend Tag-Plus enzyme (TOYOBO, Osaka, Japan) in the PCR reactions, and the operation was conducted in accordance with the instructions. A negative control was used in each PCR amplification. After amplification, 5 μL of PCR product was separated on 1% agarose gel electrophoresis and visualized by ethidium bromide staining. PCR products containing the target fragment were sent to the Nanjing GenScript Biological Technology Company for bidirectional sequencing. We conducted phylogenetic analyses based on the nucleotide sequences of *glpQ* (555 bp) using the maximum likelihood method [28] in MEGA 6.0 [29]. We searched for homologous sequences with BLAST in NCBI and downloaded them. Clustal W software was used for sequence alignment analysis, and its reliability was tested with bootstrap analysis with 1000 replicates.

### De novo sequencing and multilocus sequencing analysis based on draft genome data of cultured isolate

Genomic DNA was extracted from the *B. miyamotoi* strain Hetao-1 according to Lim et al. [30]. For genomic library construction, 1 μg of DNA was used for DNA sample preparation, and sequencing libraries were generated using the NEBNext Ultra DNA Library Prep Kit for Illumina (New England Biolabs, USA) following the manufacturer’s instructions. Briefly, the DNA sample was fragmented by sonication to approximately 350 bp, then DNA fragments were end-polished, A-tailed, and ligated with the full-length adaptor for Illumina sequencing with further PCR amplification. The PCR products were purified (AMPure XP system), and libraries were analyzed for size distribution on the Agilent 2100 Bioanalyzer and quantified using real-time PCR. The whole genome of *B. miyamotoi* strain Hetao-1 was sequenced using the Illumina NovaSeq PE150. For genome assembly, the raw data were independently assembled using SOAPdenovo v.1.0 [31], SPAdes [32], and ABySS v.2.0 [33]. The assembly results for the three software packages were integrated with CISA software [34], and the assembly result with the fewest scaffolds was selected. De novo sequencing and assembly were performed at Beijing Novogene Bioinformatics Technology Co. Ltd.

Multilocus sequencing analysis (MLSA) was performed on the draft genome sequence of strain Hetao-1 using the concatenated loci of eight genes (*clpA*, *clpX*, *nifS*, *pepX*, *pyrG*, *recG*, *rplB*, and *uvrA*) according to Margos et al. [35].

## Results

### Ticks infected with borreliae in Inner Mongolia

A total of 2656 adult *I. persulcatus* ticks were collected from the Daxingan mountains in Hulun Buir City of Inner Mongolia, China (Fig. 1). All the collected ticks were screened for borreliae DNA by qPCR targeting of *16S rDNA*. As shown in Table 1, ticks harboring *B. miyamotoi* were found from four districts: Genhe, Yakeshi, Arong Banner, and Arxan. The overall prevalence of RF borreliae, including *B. miyamotoi*, in ticks of Hulun Buir was 5.0% (134/2656). Specifically, the percentages of ticks positive for RF borreliae were 8.6% in Genhe, 5.1% in Yakeshi, 2.6% in Arong Banner, and 14.0% in Arxan (Table 1). The overall prevalence of LD borreliae was 43.8% (1164/2656), and the percentages of ticks positive for LD borreliae by district were 59.5% in Genhe, 45.0% in Yakeshi, 31.3% in Arong Banner, and 55.8% in Arxan (Table 1). Co-infection with RF and LB borreliae was observed in 46 ticks (1.7%) in Hulun Buir.

**Table 1:** Prevalence of *Borreliae* in *Ixodes persulcatus* ticks

Location in Hulun Buir	No. of ticks			RF borreliae (including *B. miyamotoi*)-positive no. (%)			LD borreliae-positive no. (%)			Co-infection no. (%)		
	Male	Female	Total	Male	Female	Total	Male	Female	Total	Male	Female	Total
Genhe	56	60	116	6 (10.7)	4 (6.7)	10 (8.6)	36 (64.3)	33 (55.0)	69 (59.5)	3 (5.4)	0	3 (2.6)
Yakeshi	1023	1090	2113	58 (5.7)	50 (4.6)	108 (5.1)	439 (42.9)	512 (47.0)	951 (45.0)	11 (1.1)	43 (3.9)	54 (1.6)
Arong Banner	217	167	384	4 (1.8)	6 (3.6)	10 (2.6)	78 (35.9)	42 (25.1)	120 (31.3)	1 (0.5)	4 (2.4)	5 (2.1)
Arxan	22	21	43	4 (18.2)	2 (9.5)	6 (14.0)	9 (40.9)	15 (71.4)	24 (55.8)	1 (4.5)	0	1 (2.3)
Total	1315	1341	2656	72 (5.5)	62 (4.6)	134 (5.0)	562 (42.6)	602 (45.0)	1164 (43.8)	16 (1.2)	47 (3.5)	63 (2.4)

### Identification of *B. miyamotoi* in ticks

**Fig. 2:**
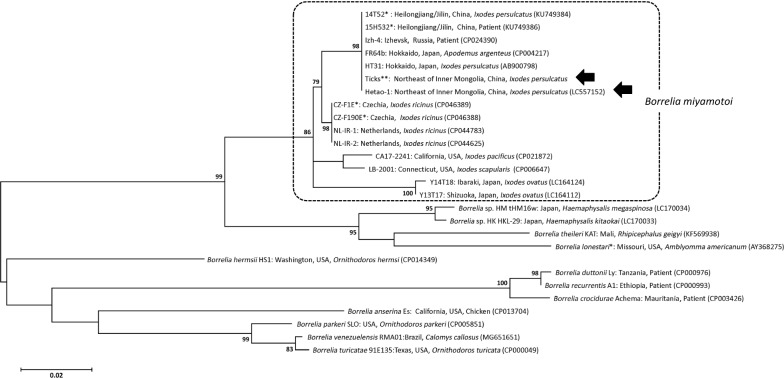
Phylogenetic analysis of RF borreliae based on *glpQ*. The tree was constructed based on *glpQ* sequences by the maximum likelihood method based on the Kimura two-parameter model with 1000-bootstrap resampling. The bar indicates the percentage of sequence divergence. *Borrelia miyamotoi* found in this study is indicated by black arrows. The origin of isolation (country and source) for each *B. miyamotoi* is listed after each lineage. The number in parentheses indicates the GenBank accession number. *Uncultured *Borrelia miyamotoi.* **Uncultured *B. miyamotoi* from *Ixodes persulcatus* in northeastern Inner Mongolia, China. Accession numbers of *B. miyamotoi* “Ticks” were from LC557142 to LC557151

To identify the RF borrelia in ticks from Hulun Buir, we performed sequence analysis followed by *glpQ* qPCR of RF-borrelia-positive samples (134 samples). Of these 134 tick samples, we successfully sequenced partial *glpQ* from 105, and these sequences were 100% identical to each another and to that of the *B. miyamotoi* strain FR64b (accession number: CP004217) (Fig. 2). Nucleotide sequences of the representative *B. miyamotoi* isolate from Hulun Buir were deposited in the DDBJ/GenBank DNA database with accession numbers LC570864–LC570882. In 29 of the tick specimens, weak or no amplification of *glpQ* was seen. The discrepancy between qPCR and *glpQ*-PCR remains unclear; however, it may be that the sensitivity of the *16S rDNA* qPCR reaction was higher than that for conventional PCR targeting the *glpQ* gene.

### Genetic characterization of *B. miyamotoi* DNA from cultured isolates and ticks using *glpQ* genes

We successfully cultured one *B. miyamotoi* isolate from an *I. persulcatus* tick using BSK-M medium. This isolate was used in the initial qPCR confirmation of the pathogen and for analyzing the *glpQ* sequences. Based on the amplified region of the *glpQ* gene, the Hetao-1 isolated in this study (accession number: LC557152) clustered together with Siberian *B. miyamotoi* strains isolated in Japan and Russia (Fig. 2).

### MLSA by draft genome sequence

**Fig. 3:**
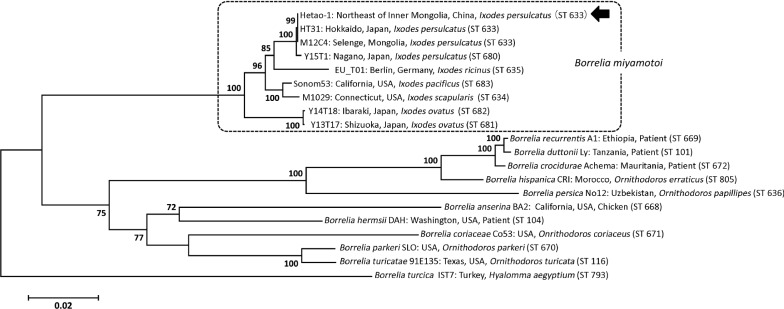
MLSA of *B. miyamotoi* Hetao-1 and other relapsing fever borreliae. A phylogenetic inference of the concatenated housekeeping gene sequences of the representative relapsing fever borreliae is shown. The arrow indicates the *B. miyamotoi* strain Hetao-1 isolated in this study. Consensus sequences for the eight housekeeping genes were isolated from the draft genome sequence of *B. miyamotoi* strains Hetao-1, trimmed to lengths and concatenated in the order: *clpA*, *clpX*, *nifS*, *pepX*, *pyrG*, *recG*, *rplB*, and *uvrA* according to the *Borrelia* PubMLST database. For phylogenetic reconstruction, the maximum likelihood model based on the Kimura two-parameter model with MEGA 6.0 was used with 1000 bootstrap replicates. *Borrelia turcica* IST7 was used as outgroup. The ST number designated in each strain indicates the “sequence type” registered in the *Borrelia* PubMLST database

A draft genomic sequence of the *B. miyamotoi* isolated from ticks sampled in Inner Mongolia was obtained to characterize the Hetao-1 strain. The chromosome of the strain was estimated to be approximately 906.1 kbp in length, with GC content of 28.9%. The chromosome sequence showed 46 single-nucleotide polymorphisms (SNPs) without Ins/Del compared with the *B. miyamotoi* strain Izh-4 (Accession number: CP024390) [36]. Using the genome assembly data, MLSA was carried out using eight genes (*clpA*, *clpX*, *nifS*, *pepX*, *pyrG*, *recG*, *rplB,* and *uvrA*) isolated from the draft genome sequence. Analysis of the eight concatenated housekeeping gene sequences (4776 nucleotides) identified the Chinese Hetao-1 isolate from *I. persulcatus* as ST633 and as identical to the *B. miyamotoi* Japanese isolate HT31 (Japan) and M12C4 (Mongolia) (Fig. 3).

### DNA accession numbers

Accession numbers obtained in this study are as follow: *B. miyamotoi* strain Hetao-1 CTP synthase gene (*pyrG*), DNA helicase gene (*recG*), ATP-dependent endopeptidase clp ATP-binding subunit gene (*clpX*), aspartyl aminopeptidase gene (*pepX*), excinuclease ABC subunit A gene (*uvrA*), cysteine desulfurase gene (*nifS*), ATP-dependent Clp protease subunit A gene (*clpA*), LSU ribosomal protein L2P gene (*rplB*), *16SrRNA*, and flagellin gene (*flaB*) are LC557142, LC557143, LC557144, LC557145, LC557146, LC557147, LC557148, LC557149, LC557150, and LC557151, respectively.

## Discussion

*Borrelia miyamotoi* is a newly described emerging pathogen. Before this study, no isolates of this spirochete from China had been cultured, and there was little information on *B. miyamotoi* infections in humans and ticks [12, 37, 38]. In this study, we detected *B. miyamotoi* in *I. persulcatus* ticks and successfully isolated a *B. miyamotoi* strain from *I. persulcatus* collected in Greater Khingan, Inner Mongolia, China. Our findings demonstrate that *B. miyamotoi* infection of *I. persulcatus* is widespread across the regions examined. Similar to Russia and Japan, *I. persulcatus* ticks in Greater Khingan, China, are abundant mainly in forested regions. To date, there have been no reports on the prevalence of *B. miyamotoi* infections in humans or ticks in Greater Khingan; however, human tick bites and tick-borne LD or tick-borne encephalitis (TBE) are known to occur frequently in this region. We conducted large-scale tick surveillance for *B. miyamotoi* in Greater Khingan, as human cases of *B. miyamotoi* infection were confirmed in Jilin and Heilongjiang, China, in 2018, and *B. miyamotoi* has previously been found in *I. persulcatus* ticks [12]. From our research, the prevalence of *B. miyamotoi* among *I. persulcatus* was shown to be approximately 5%, which is similar to the prevalence shown by the most recent study in China (approximately 3% in *I. persulcatus*) [12, 38]. Most previous reports suggest that *Ixodes* ticks are transmission vectors of *B. miyamotoi* in North America, Europe, and in other Asian countries. Our results support the hypothesis that *I. persulcatus* is an important vector of *B. miyamotoi* in Inner Mongolia, China, as well.

While other tick species (*Haemaphysalis* and *Dermacentor* ticks) have been found to carry *B. miyamotoi* in China [12, 37, 38], the potential for *Haemaphysalis* and *Dermacentor* ticks to act as vectors of *B. miyamotoi* remains unclear. *Haemaphysalis*, however, is suggested to be a vector of *Borrelia* species related to *B. theileri* in Japan [39, 40]. The *Borrelia* species (i.e., *Borrelia* sp. HL) was classified as a hard-tick-borne RF borrelia, but it is clearly distinguishable from *B. miyamotoi* by sequencing of several housekeeping genes [39]. Thus, further study may be required on the competency of these tick species as vectors of *B. miyamotoi* to assess the risk of BMD in China.

The data collated in this study provide information on the risk of *B. miyamotoi* human infection (Table 1). Additionally, we detected LD borreliae from 43.8% of *I. persulcatus* ticks, which are thought to be the vectors of *B. miyamotoi* and LD borreliae in Inner Mongolia*.* Although the prevalence of *B. miyamotoi* is lower than that of LD borreliae in Greater Khingan, *B. miyamotoi*, as a cause of fever and various other symptoms, is also a risk to public health.

It is known that *B. miyamotoi* found in Russia and other Asian countries is widely distributed in habitat areas of the *Ixodes persulcatus* tick. Using MLSA, we revealed that *B. miyamotoi* ST633, which has previously been found in Mongolia and Japan [18], is distributed in several regions of Inner Mongolia. Furthermore, the draft genome sequence revealed that the Inner Mongolia isolate has only 46 chromosomal SNPs compared with the *B. miyamotoi* strain Izh-4, although no geographical relationship was observed between these strains. This suggests that clonal expansion of *B. miyamotoi* may have occurred with the migration of vectors/reservoirs throughout Asian countries, including Russia. To resolve this question, further epidemiological studies of *B. miyamotoi* infection are required.

## Conclusion

In this study, we detected *B. miyamotoi* in *I. persulcatus* ticks from Inner Mongolia, China, and successfully isolated a strain of *B. miyamotoi*. To our knowledge, this is the first report of isolation of *B. miyamotoi* from China. Further epidemiological studies investigating the prevalence of *B. miyamotoi* and other borreliae in *I. persulcatus* ticks will provide new insights into the epidemiological aspects of *B. miyamotoi* infection in Inner Mongolia, China.

## Data Availability

The datasets used and/or analyzed in the current study are available from Gaowa on reasonable request.
